# Association Between Anxiety and Reflux Symptoms in Patients With Gastroesophageal Reflux Disease: A Prospective Cohort Study

**DOI:** 10.7759/cureus.73391

**Published:** 2024-11-10

**Authors:** Michaela Henning, Katharina Lindgen, Desiree Paul, Claudia Fuchs, Alexander Niecke, Christian Albus, Christiane Bruns, Kim Pelzner, Jessica Leers

**Affiliations:** 1 Department of Psychosomatics and Psychotherapy, University Hospital Cologne, University of Cologne, Cologne, DEU; 2 Department of Orthopedics, Trauma and Reconstructive Surgery, Sana Hospital Benrath, Düsseldorf, DEU; 3 Department of Visceral Surgery and Functional Lower GI Surgery, Evangelic Hospital Weyertal, Cologne, DEU; 4 Department of Functional Upper GI Surgery, Evangelic Hospital Kalk, Cologne, DEU; 5 Department of General, Visceral, Cancer and Transplantation Surgery, University Hospital Cologne, University of Cologne, Cologne, DEU

**Keywords:** anxiety, depression, gerd, hads, mental comorbidity, reflux, somatic stress disorder

## Abstract

Objective: The purpose of this paper is to examine the prevalence and symptom severity of anxiety and depression in patients with gastroesophageal reflux disease (GERD). The correlation between anxiety and depression with the DeMeester score is determined. GERD is a common gastrointestinal disorder that manifests as heartburn, regurgitation, retrosternal pain, cough, and dysphagia. Patients are severely affected by reflux symptoms.

Methods: We conducted a prospective cohort study of 458 consecutively enrolled patients who presented to the Department of Functional Upper GI Surgery at the University Hospital of Cologne, Germany. Patients underwent upper gastrointestinal endoscopy, 24-hour pH impedance testing, and high-resolution manometry. We determined the DeMeester score. Psychometric data were collected using the Hospital Anxiety and Depression Scale (HADS).

Results: 44.1% (n = 202) of patients had abnormally high HADS anxiety scores and 23.8% (n = 109) had abnormally high HADS depression scores (both >7). Patients without an indication for surgery (51.1%, n = 234) were the most distressed subgroup: 47.9% (n= 112) of these patients had an anxiety score of 8 or higher, and as many as 23.9% (n = 56) of these patients had an anxiety score of 11 or higher. We found a significant effect of elevated anxiety and elevated depression scores on the severity of symptoms such as heartburn and fullness, as well as an effect of elevated anxiety scores on the severity of dysphagia.

Conclusion: Patients with reflux symptoms have a high prevalence of anxiety. Anxiety and partly depression are significantly associated with the severity of reflux symptoms. An adequate multidisciplinary treatment strategy is required.

## Introduction

Gastroesophageal reflux disease (GERD) is one of the most common gastrointestinal disorders with a reported prevalence of 10% to 20% in Europe and the USA and less than 5% in Asia [1]. GERD manifests as heartburn, regurgitation, retrosternal pain, cough, and in some cases dysphagia. Possible complications of GERD include peptic stenosis and the development of Barrett’s esophagus, a condition in which esophageal squamous epithelium changes into gastric cylindrical epithelium due to chronic acid exposure. GERD can be manifested as either non-erosive (NERD) or erosive reflux disease (ERD). Following official guidelines, diagnostic workup includes upper gastrointestinal endoscopy, high-resolution manometry, and 24-hour pH-impedance testing. Important differential diagnoses of GERD are benign functional disorders of the esophagus, such as functional heartburn or functional dysphagia and esophageal motility disorders [2]. Treatment of GERD can either be conservatively or surgically. Conservative therapy includes lifestyle adjustments as well as intake of proton pump inhibitors to reduce the acidity of the reflux. In contrast, antireflux surgery is performed to correct the anatomy of the esophagogastric junction in order to provide a functional barrier that prevents pathological amounts of gastric acid from entering the esophagus. Antireflux surgery is a successful option for patients with persistent symptoms and/or progression of disease despite appropriate drug treatment, complicated GERD, or permanently impaired quality of life.

Comorbid symptoms of anxiety and depression are common in GERD patients. In previous studies, the association between anxiety or depression and reflux symptoms has been thoroughly investigated. It was examined whether the presence of reflux symptoms leads to an increased level of anxiety or depression and whether anxiety and depression both lead to an increased symptom perception. In GERD patients, the prevalence of anxiety, depression, and sleep disturbances is higher than in those without GERD [3]. Patients with anxiety are more likely to experience GERD symptoms, and these symptoms seem to be more severe in anxious or depressed patients [4]. Furthermore, there is an independent association between GERD, anxiety, and current depression [5], and GERD is considered to be an independent source of stress [6].

A long duration of GERD is associated with higher levels of anxiety and depression, and women are more likely to experience these symptoms [7]. In patients with Barrett's esophagus, rates of anxiety and depression have been reported to be three to five times higher than in the general population [8].

Anxiety and depression as well as adverse events in life are independent risk factors for NERD [9]. Patients with NERD show an increased risk for anxiety compared with patients with ERD [8]. All in all, there is an interaction between GERD and mental disorders [10], which has been described as the "psychoemotional effects of GERD" [7].

The reporting of somatic symptoms is multifactorial and influenced by psychosocial factors, such as socioeconomic status, sex, and mental distress [10,11]. A high somatic symptom load is known to increase anxiety related to health issues, psychological distress, and health care utilization [12,13]. Anxiety and depression also play a role in an increased sensitivity to visceral stimuli, which has been described as visceral hypersensitivity [14].

The aim of this study was to assess the anxiety and depression levels of patients who presented with reflux symptoms at our clinic. We further examined the impact of anxiety on the severity of reflux symptoms such as heartburn, fullness, and dysphagia.

## Materials and methods

Ethics statement

The present study was conducted according to the Declaration of Helsinki principles and was approved by the Ethics Committee of the Medical Faculty of the University of Cologne (Approval No. 19-1495). All patients provided their online informed consent. The study has been registered at ClinicalTrials.gov (NCT06151067).

Study design

We conducted a prospective, monocentric, observational cohort study with a single measurement time point during the diagnostic evaluation.

Participants

Between January 2020 and July 2021, all patients who presented at the Department of Functional Upper GI Surgery of the Clinic for General, Visceral, Cancer and Transplantation Surgery of the University Hospital Cologne in Germany, were included in the study. All patients underwent upper gastrointestinal endoscopy, 24-hour pH-impedance testing, and high-resolution manometry as a diagnostic workup. Psychometric data were collected using the Hospital Anxiety and Depression Scale (HADS) (German version) questionnaire [15]. All patients who did not fill in the HADS questionnaire were excluded from the study.

Indication for antireflux surgery was given in case of (a) a pathological DeMeester score, (b) the presence of hiatal hernia, (c) the presence of reflux esophagitis LA-grade C/D, or (d) the presence of GERD complications such as peptic stenosis or Barrett’s esophagus, as well as (e) presence of limiting reflux symptoms. Not all the above-mentioned criteria needed to be fulfilled, in order to be considered for antireflux surgery. Additional criteria such as poor response to medication as well as decreased health-related quality of life due to a high symptom burden, were also taken into account during the assessment.

Questionnaires

The severity of reflux symptoms as well as anxiety and depression levels were determined using standardized, validated questionnaires, including the Hospital Anxiety and Depression Scale (HADS) [15] as well as the GERD-Health Related Quality of Life questionnaire (GERD-HRQL) [16].

The HADS (German version) was used for the assessment of anxiety (HADS-A) and depression levels (HADS-D). Accordingly, patients self-reported anxiety and depression levels during the previous week before the diagnostic workup. The questionnaire consists of two subscales (anxiety and depression) with seven items each, ranging from 0 - 3 points, adding up to a total score of 21. For both subscales, a cutoff score for caseness of > 8 is recommended [17].

For the evaluation of HADS scores, subscales of ≥ 8 were labeled as either “anxious” or “depressed”. It was differentiated between mildly (score 8-10), moderately (score 11 - 14) and severely (score 15 - 21) anxious or depressed.

The GERD-HRQL questionnaire is a standardized instrument to measure the severity of symptoms in GERD patients. It consists of 10 items on specific GERD symptoms, such as heartburn, fullness, or dysphagia. Items can be scored between 0 (= no symptoms) and 5 (= symptoms are incapacitating - unable to do daily activities).

Twenty-four-hour pH-impedance testing

Pathological acid exposure was determined using 24-hour pH-impedance testing, a method, in which gastric acid exposure of the distal esophagus is measured during a period of 24 hours, using a thin flexible catheter that is placed into the esophagus.

Afterward, the DeMeester score was determined, based on the following measurement values: percentage of time with esophageal pH < 4 of the total measurement period, percentage of time with pH < 4 during the waking phase (upright position), percentage of time with pH < 4 during sleep phase (supine position), total number of reflux episodes during the measurement period, number of reflux episodes with duration > 5 min and duration of the longest reflux episode.

A DeMeester score equal to or below 14.72 was interpreted as physiological, whereas a score above 14.72 indicated a pathological gastric acid exposure of the distal esophagus. Pathological scores were differentiated into mild (14.72 - 30), moderate (30 - 80), and severe (> 80).

Statistical analysis

Statistical analysis was performed using IBM SPSS Statistics for Windows, Version 28.0.1 (IBM Corp., Armonk, NY, USA). Continuous variables were presented as mean and standard deviation and categorical data as frequencies with percentages. To identify differences between the two groups, an independent samples T-test was performed for parametric data, whereas a Mann-Whitney U test was performed for non-parametric data. In order to evaluate the relationships between categorical variables, we employed Pearson's Chi-square test. We elected to exclude the continuity correction, as our sample size was sufficient to satisfy the assumptions necessary for this test, thereby ensuring the reliability of our findings. To address the potential for false positives resulting from multiple comparisons, we applied a False Discovery Rate (FDR) correction using the Benjamini-Hochberg procedure to all p-values obtained from the Chi-square tests, t-tests, and Mann-Whitney U tests. We chose this method because it offers greater statistical power compared to other correction techniques, such as the Bonferroni correction, especially in exploratory studies that involve testing multiple hypotheses. Correlation between parameters was investigated using Pearson's correlation coefficient for parametric data and Spearman's correlation coefficient for non-parametric data. A p-value < 0.05 was considered statistically significant.

## Results

A total of 458 patients were included in the study (mean age of 55 years, ranging from 18 to 86, with 168 male patients). In all, 257 patients completed both HADS- and GERD-HRQL questionnaires, and in 449 patients a DeMeester score was determined (Table 1).

**Table 1:** Patient demographics. HADS: Hospital Anxiety and Depression Scale, GIQLI: Gastrointestinal Quality of Life Index.

	Total	DeMeester < 14.72	DeMeester > 14.72	p-value
Total number of patients, n (%)	449	169 (38)	280 (62)	
Women, n (%)	281 (62.6)	116 (68.6)	165 (58.9)	0.059
Men, n (%)	168 (37.4)	53 (31.4)	115 (41.1)	
Characteristics				
Mean age, yrs ± SD	55 ± 15	53 ± 15.6	54.4 ± 14.5	0.504
Mean BMI, kg/m2 ± SD	28.4 ± 5.1	28.1 ± 5	28.6 ± 5.4	0.602
Smoking, n (%)	62 (13.8)	14 (8.2)	48 (17.1)	0.039
Alcohol consumption, n (%)	124 (27.6)	45 (26.6)	79 (28.2)	0.983
pH-Impedance testing				
DeMeester score, mean ± SD	41.3 ± 123.8	6.6 ± 4.1	62.2 ± 153	0.004
Health-related quality of life				
HADS total, mean score ± SD	12.4 ± 7.4	12.1 ± 7	12.7 ± 7.7	0.504
Anxiety, mean score ± SD	7.2 ± 4.2	7.4 ± 4.1	7.1 ± 4.3	0.504
Depression, mean score ± SD	5.2 ± 4.1	4.7 ± 3.9	5.6 ± 4.2	0.602
GIQLI, mean score ± SD	89.6 ± 22.6	93.5 ± 20.2	87.1 ± 23.5	0.011

An elevated HADS-A score was shown in 202 patients (44.1%). Women had a significantly higher HADS-A score than men (48.9%, n= 139, vs. 36.2%, n = 63, p = 0.027). No significant difference between female and male patients was found regarding HADS-D scores (23.9%, n = 68, vs. 23.6%, n = 41, p = 0.734) (Table 2).

**Table 2:** HADS scores for respective patient groups. HADS: Hospital Anxiety and Depression Scale.

	Total N	Female	Male	p-value	Total N	DeMeester < 14.72	DeMeester > 14.72	p-value	Total N	No surgery indication	Surgery indication	p-value
Number of patients, n (%)	458	284 (62)	174 (38)		437	167 (38.2)	270 (61.8)		455	234 (51.2)	221 (48.8)	
HADS total score, mean ± SD		13 ± 7.7	11.4 ± 6.8	0.048		12.1 ± 7	12.7 ± 7.7	0.540		12.8 ± 7.6	12 ± 7.1	0.378
HADS anxiety score, mean ± SD		7.7 ± 4.2	6.5 ± 4	0.009		7.4 ± 4.1	7.1 ± 4.3	0.602		7.6 ± 4.4	6.8 ± 4	0.123
Not anxious (score 0-7), n (%)	256	145 (51.1)	111 (63.8)	0.027	244	89 (53.3)	155 (57.4)	0.565	253	122 (52.1)	131 (59.3)	0.450
Anxious (score 8-21), n (%)	202	139 (48.9)	63 (36.2)	0.027	193	78 (46.7)	115 (42.6)	0.565	202	112 (47.9)	90 (40.7)	0.450
Mildly anxious (score 8-10), n (%)	108	79 (56.8)	29 (46)	0.027	104	43 (55.1)	61 (53)	0.565	108	56 (50)	52 (57.8)	0.940
Moderately anxious (score 11-14), n (%)	65	37 (26.6)	28 (44.5)	0.603	60	26 (33.4)	34 (29.6)	0.565	65	39 (34.8)	26 (28.9)	0.450
Severely anxious (score 15-21), n (%)	29	23 (16.5)	6 (9.5)	0.118	29	9 (11.5)	20 (17.4)	0.565	29	17 (15.2)	12 (13.3)	0.940
HADS depression score, mean ± SD		5.3 ± 4.2	5 ± 3.9	0.351		4.7 ± 3.9	5.6 ± 4.2	0.087		5.2 ± 4.1	5.2 ± 4.1	0.967
Not depressed (score 0-7), n (%)	349	216 (76.1)	133 (76.4)	0.734	334	136 (81.4)	198 (73.3)	0.968	347	180 (76.9)	167 (75.6)	0.940
Depressed (score 8-21), n (%)	109	68 (23.9)	41 (23.6)	0.734	103	31 (18.6)	72 (26.7)	0.968	108	54 (23.1)	54 (24.4)	0.940
Mildly depressed (score 8-10), n (%)	53	33 (48.5)	20 (48.8)	0.940	49	17 (54.8)	32 (44.4)	0.968	53	27 (50)	26 (48.2)	0.940
Moderately depressed (score 11-14), n (%)	39	22 (32.4)	17 (41.5)	0.601	37	8 (25.8)	29 (40.3)	0.644	38	18 (33.3)	20 (37)	0.940
Severely depressed (score 15-21), n (%)	17	13 (19.1)	4 (9.7)	0.899	17	6 (19.4)	11 (15.3)	0.422	17	9 (16.7)	8 (14.8)	0.940

Table 2 shows HADS scores of different subgroups, including sex, pathological, and physiological DeMeester scores as well as patients with and without a surgery indication. Patients with a physiological DeMeester score showed a higher mean HADS-A score compared to patients with a pathological DeMeester score (7.4 ± 4.1 vs. 7.1 +/- 4.3, p = 0.6). The latter also showed a higher HADS-D score (5.6 ± 4.2 vs. 4.7 ± 3.9, p = 0.087) compared to patients with a physiological DeMeester score, however, both groups remained below the cut-off value.

A total of 221 patients were eligible for antireflux surgery. Patients without a surgery indication had higher HADS-A scores compared to patients with an indication for surgery (6.8 ± 4.0 vs. 7.6 ± 4.4, p = 0.123).

In Table 3 respective HADS scores for patients with mild, moderate, and severe esophageal acid exposure are shown. The results did not show any significant differences between the subgroups.

**Table 3:** HADS scores for respective DeMeester subgroups, including mild, moderate, and severe esophageal acid exposure. HADS: Hospital Anxiety and Depression Scale.

	DeMeester subgroups				
	Negative	Mild	Moderate	Severe	p-value
Total number of patients, n (%)	167 (38.2)	103 (23.6)	117 (26.8)	50 (11.4)	
HADS total score, mean ± SD	12.06 ± 7.03	13.54 ± 8.2	12.22 ± 7.09	12.32 ± 8	0.438
HADS anxiety score, mean ± SD	7.35 ± 4.12	7.64 ± 4.59	6.74 ± 3.76	7.04 ± 4.66	0.418
Not anxious (score 0-7), n (%)	89 (53.3)	58 (56.3)	70 (59.8)	27 (54)	0.818
Anxious (score 8-21), n (%)	78 (46.7)	45 (43.7)	47 (40.2)	23 (46)	0.818
Mildly anxious (score 8-10), n (%)	43 (55.1)	16 (35.6)	30 (63.8)	15 (65.2)	0.345
Moderately anxious (score 11-14), n (%)	26 (33.4)	21 (46.6)	12 (25.5)	1 (4.3)	0.100
Severely anxious (score 15-21), n (%)	9 (11.5)	8 (17.8)	5 (10.6)	7 (30.5)	0.345
HADS depression score, mean ± SD	4.67 ± 3.91	5.71 ± 4.14	5.53 ± 4.24	5.28 ± 4.14	0.161
Not depressed (score 0-7), n (%)	136 (81.4)	77 (74.8)	86 (73.5)	35 (70)	0.403
Depressed (score 8-21), n (%)	31 (18.6)	26 (25.2)	31 (26.5)	15 (30)	0.403
Mildly depressed (score 8-10), n (%)	17 (54.8)	9 (34.6)	14 (45.2)	9 (60)	0.519
Moderately depressed (score 11-14), n (%)	8 (25.8)	13 (50)	12 (38.7)	4 (26.7)	0.345
Severely depressed (score 15-21), n (%)	6 (19.4)	4 (15.4)	5 (16.1)	2 (13.3)	0.993

With respect to Table 4, patients with elevated anxiety scores reported a significantly higher severity of symptoms, such as heartburn (p = 0.002), dysphagia (p = 0.038), and fullness (p = 0.003), compared to patients without elevated HADS-A values. The same was observed with respect to HADS-D scores on the severity of heartburn (p > 0.002) and fullness (p = 0.015); however, the severity of dysphagia was not significantly different between the groups.

**Table 4:** Severity of reflux symptoms in relation to different levels of anxiety and depression. HADS: Hospital Anxiety and Depression Scale, HADS-A: HADS-subscale anxiety, HADS-D: HADS-subscale depression.

	HADS-A < 8	HADS-A > 8	U-value	p-value	HADS-D < 8	HADS-D > 8	U-value	p-value
Total number of patients, n (%)	146 (59.1)	101 (40.9)			193 (78.1)	54 (21.9)		
Symptoms								
Heartburn, mean ± SD	2.28 ± 1.39	2.96 ± 1.39	5344.0	0.002	2.37 ± 1.39	3.22 ± 1.36	3376.5	0.002
Dysphagia, mean ± SD	1.35 ± 1.46	1.76 ± 1.44	6179.5	0.038	1.45 ± 1.42	1.79 ± 1.59	4607.0	0.120
Fullness, mean ± SD	2.08 ± 1.41	2.66 ± 1.47	5495.0	0.003	2.19 ± 1.46	2.77 ± 1.38	3874.5	0.015

Furthermore, a significant correlation between the DeMeester score and HADS-A or HADS-D scores could not be found (Table 5).

**Table 5:** Correlation between the DeMeester score and HADS-A and HADS-D scores. HADS: Hospital Anxiety and Depression Scale, HADS-A: HADS-subscale anxiety, HADS-D: HADS-subscale depression.

	HADS total		HADS-A		HADS-D	
	Correlation coefficient	p-value	Correlation coefficient	p-value	Correlation coefficient	p-value
DeMeester score	0.15	0.760	-0.24	0.623	0.57	0.235

Our patients had a higher prevalence of anxiety compared to healthy subjects, cardiological inpatients and outpatients, as well as oncological outpatients. A comparison between our patients and the results presented by Herrmann-Lingen et al. [15] can be found in Table 6.

**Table 6:** Comparison of mean HADS scores between gastroesophageal reflux disease (GERD) patients, cardiological and oncological patients as well as a healthy control group as presented by Hermann-Lingen et al. [15]. Results of cardiological and oncological patients as well as of a healthy control group quoted as presented by Herrmann-Lingen et al. [15]. HADS: Hospital Anxiety and Depression Scale, HADS-A: HADS-subscale anxiety, HADS-D: HADS-subscale depression.

		N	%	Age (years)	HADS-A	HADS-D
Our patients	All	458	100	54.2 +/- 14.3		
	Men	174	38	53.3. +/- 14.7	6.5 +/- 4.0	5.0 +/- 3.9
	Women	284	62	54.8 +/- 15.0	7.7 +/- 4.2	5.3 +/- 4.2
Cardiology patients (in and outpatients) [15]	All	5579	100	42.0 +/- 15.4	6.8 +/- 4.1	5.0 +/- 3.7
	Men		74		6.4 +/- 4.0	4.9 +/- 3.7
	Women		26		7.7 +/- 4.1	5.4 +/- 3.8
Oncology patients [15]	All	77	100	42.0 +/-15.4	5.9 +/- 3.7	5.4 +/- 4.6
	Men		38		5.8 +/- 3.8	5.2 +/- 4.9
	Women		62		6.1 +/- 3.5	5.7 +/- 4.1
Healthy control group [15]	All	152	100	42.0 +/-15.4	5.8 +/-3.2	3.4 +/- 2.6
	Men	25	38		5.1 +/- 3.0	3.7 +/- 2.7
	Women	127	62		6.3 +/- 3.2	3.2 +/- 2.6

## Discussion

The present study investigated the prevalence of anxiety and depression in patients with reflux symptoms. A high prevalence of anxiety was found in reflux patients, in both men and women. On average, women had a significantly higher HADS-A score compared to men. Compared to other studies on reflux symptoms and mental comorbidities that also used the HADS to assess depression and anxiety by Kessing et al., Boltin et al., Kim et al., and Neto et al., our results are in concordance with respective outcomes. On average HADS-A scores ranged between 6.0 and 8.5 [4,18-20].

Compared to the results of Hermann-Lindgen et al., our patients had a higher prevalence of anxiety compared to healthy subjects, cardiological inpatients and outpatients, as well as oncological outpatients [15]. 

Furthermore, a high incidence of depression was found in the present study. Compared to the literature, HADS-D scores are found approximately in the same range, on average between 3.5 and 8.0 [4,18-20]. Regarding depression, only oncological outpatients showed a higher median HADS-D score [15].

In the present study, a correlation between HADS-A and HADS-D scores with the DeMeester score could not be found. To our knowledge, only Neto et al. investigated this very research question [20]. In both studies, a correlation between the DeMeester score with the HADS total score as well as the HADS-D score could not be found. However, regarding anxiety levels, the results are different. Our current study did not find a correlation between anxiety and DeMeester scores, whereas the results of Neto et al. indicated a negative correlation.

The present study further showed higher anxiety levels in patients with physiological DeMeester scores and in patients without a surgery indication. These results may indicate that anxiety increases the perception of symptoms [4]. On the other hand, it may suggest that the treating physician is more hesitant to recommend antireflux surgery to those patients with a high level of anxiety, who may also pronounce doubts or ambivalence when it comes to surgery.

The results further showed a significantly higher severity of reflux symptoms in patients with elevated anxiety and depression levels. This could be understood in two ways: on the one hand, patients with a high somatic symptom load suffer from higher levels of anxiety and depression [15]. On the other hand, it implies that anxiety and depression might modulate the severity of reflux symptoms. This is consistent with previous findings. The literature suggests that patients with anxiety are more likely to experience GERD symptoms and that these symptoms seem to be more severe in anxious patients [4]. Moreover, it has been suggested that anxiety and depression intensify symptom perception [21].

Our patients suffered from an impaired health-related quality of life with an on average reduced GIQLI (Gastrointestinal Quality of Life Index) score to 62% of the maximum possible which is in accordance with the described range of these patient groups of 55-75% of the maximum possible in previous studies [22].

Patients with reflux symptoms have a high prevalence of anxiety and anxiety and partly depression are associated with the severity of reflux symptoms. Whether this is a manifestation of a somatic stress disorder requires multidisciplinary assessment. An appropriate multidisciplinary treatment strategy for these patients is important. A possible treatment option for patients with anxiety disorders and somatic comorbidities was presented by Henning et al. with a strong therapy focus on a biopsychosocial model of the disease [23].

This study has potential limitations. This is a single-center study, although we have a large sample size, and we only have one measurement time. Our results were obtained exclusively from the evaluation of self-report questionnaires regarding patients’ mental health burden, and only depression- and anxiety-related scores were collected. We did not have any information on possible pre-diagnosed mental illness. We only evaluated symptom scores and did not systematically collect diagnostic criteria for possible existing mental illness. Future studies should consider the use of standardized diagnostic interviews and consider other mental illnesses besides anxiety and depression, such as somatic stress disorder. Given the exploratory nature of our study, the HADS scores were repeatedly used for several variables and interpreted so that alpha inflation needed to be corrected. In future research, a more targeted approach may help minimize the need for corrections due to multiple testing, thereby facilitating a more differentiated interpretation of the results.

## Conclusions

In conclusion, our results show that patients with reflux symptoms have a high prevalence of anxiety. Anxiety and partly depression are associated with the severity of reflux symptoms. Therefore, a thorough multidisciplinary evaluation is recommended to determine whether the association of reflux symptoms with anxiety and depression is a manifestation of a somatic stress disorder. Finally, an appropriate multidisciplinary treatment strategy for these patients is of great importance.

## References

[1] El-Serag HB, Sweet S, Winchester CC, Dent J (2014). Update on the epidemiology of gastro-oesophageal reflux disease: a systematic review. Gut.

[2] Kumar AR, Katz PO (2013). Functional esophageal disorders: a review of diagnosis and management. Expert Rev Gastroenterol Hepatol.

[3] Bai P, Bano S, Kumar S (2021). Gastroesophageal reflux disease in the young population and its correlation with anxiety and depression. Cureus.

[4] Kessing BF, Bredenoord AJ, Saleh CM, Smout AJ (2015). Effects of anxiety and depression in patients with gastroesophageal reflux disease. Clin Gastroenterol Hepatol.

[5] On ZX, Grant J, Shi Z (2017). The association between gastroesophageal reflux disease with sleep quality, depression, and anxiety in a cohort study of Australian men. J Gastroenterol Hepatol.

[6] Choi JM, Yang JI, Kang SJ (2018). Association between anxiety and depression and gastroesophageal reflux disease: results from a large cross-sectional study. J Neurogastroenterol Motil.

[7] Kamolz T, Velanovich V (2002). Psychological and emotional aspects of gastroesophageal reflux disease. Dis Esophagus.

[8] Hinz A, Ernst J, Glaesmer H, Brähler E, Rauscher FG, Petrowski K, Kocalevent RD (2017). Frequency of somatic symptoms in the general population: Normative values for the Patient Health Questionnaire-15 (PHQ-15). J Psychosom Res.

[9] Chen LP, Huang ZW, Xiao B (2016). [Risk factors and clinical characteristics of gastroesophageal reflux disease: analysis based on a prospective database of functional gastrointestinal disease]. Nan Fang Yi Ke Da Xue Xue Bao.

[10] He M, Wang Q, Yao D, Li J, Bai G (2022). Association between psychosocial disorders and gastroesophageal reflux disease: a systematic review and meta-analysis. J Neurogastroenterol Motil.

[11] Beutel ME, Wiltink J, Ghaemi Kerahrodi J (2019). Somatic symptom load in men and women from middle to high age in the Gutenberg Health Study - association with psychosocial and somatic factors. Sci Rep.

[12] Creed FH, Davies I, Jackson J (2012). The epidemiology of multiple somatic symptoms. J Psychosom Res.

[13] Lee S, Creed FH, Ma YL, Leung CM (2015). Somatic symptom burden and health anxiety in the population and their correlates. J Psychosom Res.

[14] Losa M, Manz SM, Schindler V, Savarino E, Pohl D (2021). Increased visceral sensitivity, elevated anxiety, and depression levels in patients with functional esophageal disorders and non-erosive reflux disease. Neurogastroenterol Motil.

[15] Herrmann-Lingen C, Buss U, Snaith R (2018). Hospital Anxiety and Depression Scale (HADS-D): Deutsche version. Deutschsprachige Adaptation der Hospital Anxiety.

[16] Velanovich V (2007). The development of the GERD-HRQL symptom severity instrument. Dis Esophagus.

[17] Bjelland I, Dahl AA, Haug TT, Neckelmann D (2002). The validity of the Hospital Anxiety and Depression Scale: an updated literature review. J Psychosom Res.

[18] Boltin D, Boaz M, Aizic S, Sperber A, Fass R, Niv Y, Dickman R (2013). Psychological distress is not associated with treatment failure in patients with gastroesophageal reflux disease. J Psychosom Res.

[19] Kim JY, Kim N, Seo PJ (2013). Association of sleep dysfunction and emotional status with gastroesophageal reflux disease in Korea. J Neurogastroenterol Motil.

[20] Neto RM, Herbella FA, Zugman A, Velanovich V, Montera B, Schlottmann F, Patti MG (2019). Minor psychiatric disorders and objective diagnosis of gastroesophageal reflux disease. Surg Endosc.

[21] Lapina NS, Borovkov NN (2008). Anxious depressive conditions in patients with gastroesophageal reflux disease (Article in Russian). Klin Med (Mosk).

[22] Fuchs KH, Musial F, Eypasch E, Meining A (2022). Gastrointestinal quality of life in gastroesophageal reflux disease: a systematic review. Digestion.

[23] Henning M, Subic-Wrana C, Wiltink J, Beutel M (2020). Anxiety disorders in patients with somatic diseases. Psychosom Med.

